# Supplementary material for: Differential modulation of haematopoietic and oxidative injury by PARP-1 and ATR kinase inhibition in a murine model of acute irradiation
Source: PLoS One. 2026 Mar 6;21(3):e0344101. doi: 10.1371/journal.pone.0344101 (PMC12965550; doi:10.1371/journal.pone.0344101)
Supplement: Attachment — Submitted filename: respon.docx [file pone.0344101.s003.docx]

**Response to Reviewers – PONE-D-25-44594R1**

**Dear Academic Editor Dr. Hesham M.H. Zakaly and Esteemed Reviewers,**

Thank you for your thoughtful and constructive feedback on our manuscript, “Mechanisms of Cellular Repair Following Acute Nuclear Radiation Exposure: Implications for Therapy.” We have thoroughly revised the manuscript, now titled **“Differential Modulation of Haematopoietic and Oxidative Injury by PARP-1 and ATR Kinase Inhibition in a Murine Model of Acute Irradiation,”** to address all concerns raised. We believe the revisions have significantly strengthened the study’s clarity, statistical rigor, and mechanistic depth.

Below, we provide a detailed, point-by-point response to each comment. All changes in the manuscript are highlighted in the ‘Revised Manuscript with Track Changes’ file.

**RESPONSES TO ACADEMIC EDITOR’S COMMENTS**

**EDITOR COMMENT 1:**
*“The tables, while data-rich, are currently difficult to interpret. The presentation of p-values for all groups against the control, without a clear indication of inter-group comparisons (e.g., Radiation Only vs. Radiation + PARPi), weakens the statistical narrative… The statistical analysis should be refocused. Please include direct pairwise comparisons between critical experimental groups… Consider using subscript letters in the tables to denote significant differences…”*

**OUR RESPONSE:**
We thank the Editor for this crucial observation. To address this, we have **completely restructured our statistical presentation and data tables.** The revised manuscript now includes a new suite of **comparative tables (Tables 1A/B, 2A/B, 3A/B, 4A/B, 5A/B)** that are dedicated to direct pairwise analysis. These tables provide:

- **Percentage Change Analysis:** Showing % difference versus control and versus the Radiation Only group.
- **Direct Statistical Comparisons:** Clear p-values for key comparisons (e.g., Radiation Only vs. Radiation + PARPi).
- **Biological Interpretation:** A column summarizing the clinical/biological significance of each difference.
  Furthermore, in the results text, we now explicitly state the outcomes of these critical comparisons. For example:
- The combination of PARP inhibition with radiation attenuated the decrease in WBC counts to 19.23%, which was significantly less severe than the 32.31% reduction observed in the radiation-only group (p=0.008; Table A1).
- The Radiation + ATR group showed no significant protection (p = 0.812).
  This new format ensures the statistical narrative is sharp, focused, and directly supports our main conclusions.

**EDITOR COMMENT 2:**
*“A central point of confusion is the dual nature of the PARPi results… This paradox is not sufficiently explored. So, the discussion must be expanded to offer a coherent hypothesis for these divergent effects.”*

**OUR RESPONSE:**
We agree that this is the most intriguing finding of our study. We have substantially **expanded the Discussion sections (particularly in sections 3.1.1, 3.3.1, and the integrated discussion in 3.1.2)** to provide a unified and coherent mechanistic hypothesis.

We propose that **delayed PARP inhibition does not primarily accelerate the core DNA double-strand break (DSB) repair machinery.** Instead, its protective effect stems from **interrupting a secondary, maladaptive signalling cascade** triggered by excessive PARP-1 activation after irradiation. This cascade involves:

1. **NAD⁺ and ATP depletion**, leading to a metabolic crisis (parthanatos).
2. **Mitochondrial dysfunction**, which amplifies reactive oxygen species (ROS) generation.
3. **Sustained DDR signaling (prolonged γ-H2AX)** that becomes uncoupled from the original DNA lesions.

By inhibiting PARP-1 shortly after radiation, we **preserve cellular energy homeostasis and mitigate secondary oxidative stress**. This explains:

- **Why oxidative markers improve (↓MDA, ↑SOD):** The feed-forward loop of ROS production is broken.
- **Why hematological parameters are partially protected:** Hematopoietic stem/progenitor cells are spared from metabolic catastrophe.
- **The nuanced γ-H2AX data:** The reduction in *late* γ-H2AX foci with PARPi reflects dampened persistent signaling, not necessarily faster ligation of DSBs. We now carefully discuss this distinction, citing literature on γ-H2AX persistence (e.g., Bourton et al., *Radiat Res* 2012).

This reframes our results from a “contradiction” to a **revealing insight into the temporal duality of the DDR**: an early phase focused on physical DNA repair and a later phase driven by metabolic and signaling stress, which can be selectively modulated.

**EDITOR COMMENT 3:**
*“The manuscript would benefit from a thorough proofread by a native English speaker to improve sentence fluency and correct minor grammatical inconsistencies…”*

**OUR RESPONSE:** The entire manuscript has undergone **comprehensive language editing and proofreading.** We have corrected grammatical errors (e.g., “radiodensities” is now “radiosensitizes”), standardized spelling (to British English: haematological, labelled), and improved sentence structure for clarity and fluency. We are confident the manuscript now meets the high language standards of PLOS ONE.

**EDITOR COMMENT 4:** *“The transition between sections, particularly from Results to Discussion, can be abrupt. Some results are discussed within the ‘Results’ section, while others are saved for the ‘Discussion.’ Consider consolidating all interpretations into the Discussion for a cleaner structure.”*

**OUR RESPONSE:** We have reorganized the flow for better clarity. The **Results section (Section 3)** now presents the data objectively with essential statistical commentary but reserves broader mechanistic synthesis for the **Discussion.**
To create a smoother transition, we have integrated a **“Results and Discussion”** approach for key subsections (e.g., 3.1.1, 3.2.1), where immediate interpretation of the data is necessary for understanding. However, the overarching mechanistic model, comparison with prior literature, and translational implications are consolidated into the expanded narrative within the Results and the final **Conclusion section**, providing a clean, logical progression from data presentation to synthesis.

**EDITOR COMMENT 5 (Implicit – Elevating the Narrative):**
*“The authors' primary challenge will be elevating the narrative from a ‘data dump’ to an insightful mechanistic story… if the authors can successfully reframe this… the impact of the paper will be significantly enhanced.”*

**OUR RESPONSE:** We sincerely thank the Editor for this guiding suggestion. It has been central to our revision. We have consciously shifted the manuscript’s narrative from a descriptive report to a **hypothesis-driven story about temporal modulation.**

- **The new title** reflects this focus on *differential* and *modulation*.
- **The rewritten Introduction** sets up the critical question of *delayed intervention*.
- **The results are framed** to highlight the *contrast* between PARP and ATR inhibition.
- **The expanded Discussion** explicitly presents the **“temporal duality” model** of the DDR, positioning our findings as a valuable insight for designing smarter radioprotection strategies that separate anti-tumor radiosensitization from normal tissue protection. We believe the revised manuscript now offers a clear, impactful mechanistic story that aligns with the journal’s scope.

**RESPONSES TO REVIEWER #1’S COMMENTS**

**REVIEWER COMMENT 1:**
*“Please revise the language carefully.”*

**OUR RESPONSE:** Done. As detailed in response to the Editor’s Comment 3, the entire manuscript has been meticulously proofread and edited for language clarity and correctness.

**REVIEWER COMMENT 2:** *“You must state the main result in the abstract.”*

**OUR RESPONSE:** The **Abstract has been completely rewritten.** It now clearly states the main experimental finding and mechanistic conclusion:
*“Conclusion: While administering PARP inhibitors following the exposure to ionising radiation was found to reduce the severity of the condition… the same was not seen when the treatment with ATR inhibitors was given… The results of this study show the significance of administering therapeutic agents at the right time…”*

**REVIEWER COMMENT 3:**
*“The title must be revised according to the main contribution and approach of this study.”*

**OUR RESPONSE:** We have changed the title to:
**“Differential Modulation of Haematopoietic and Oxidative Injury by PARP-1 and ATR Kinase Inhibition in a Murine Model of Acute Irradiation”**
This new title accurately captures the comparative nature of the study, the key pathways investigated, the main injury endpoints measured, and the model used.

**REVIEWER COMMENT 4:**
*“The abstract is generally weak and lacks perspectives regarding the obtained results for the intended application. Please improve the wording in this section.”*

**OUR RESPONSE:** Thank you. The revised Abstract now includes a specific perspective on application:
*“The results of this study show the significance of administering therapeutic agents at the right time to stop the injury to normal cells when DNA repair pathways are activated by exposure to ionising radiation.”*
This directly links the findings to potential therapeutic timing strategies.

**REVIEWER COMMENT 5:** *“The introduction must be extended with new references for different materials.”*

**OUR RESPONSE:** The **Introduction has been significantly expanded (now ~1000 words)**, providing a deeper background on the DDR, γ-H2AX kinetics, PARP/ATR biology, and the rationale for post-exposure inhibition. We have added **numerous recent and relevant references** (e.g., Li et al., *Signal Transduct Target Ther* 2023; Pilié et al., *Nat Rev Clin Oncol* 2019; Fouquerel et al., *DNA Repair* 2014) to strengthen the foundation of our study.

**REVIEWER COMMENT 6:***“What kind of applications do you consider?”*

**OUR RESPONSE:** We now explicitly discuss potential applications in the revised manuscript:

- **In the Discussion:** We mention the concept of a “rational treatment paradigm” involving early DDR inhibition for tumor radiosensitization followed by delayed intervention to protect normal tissues.
- **In the Conclusion:** We state, *“The study proves temporal modulation functions as a critical component for DDR-based therapeutic methods while showing that PARP inhibitors can create a versatile treatment method which enhances radiation therapy effectiveness against cancer and safeguards normal tissues from harm.”*
  The primary application envisaged is in **improving the therapeutic ratio of radiotherapy.**

**REVIEWER COMMENT 7:***“What are the sensitivities or precisions of the different approaches employed in this work?”*

**OUR RESPONSE:** We have added relevant methodological details to the **Materials and Methods (Section 2.7)**:

- **Hematology:** Analyzer model (Sysmex XP-300) stated.
- **Oxidative Stress Assays:** Reference to standard methods (Ohkawa et al. for MDA; McCord & Fridovich for SOD).
- **γ-H2AX Immunofluorescence:** Specific antibody (Cell Signaling, 1:500), microscope (Leica DMi8), and quantification protocol (>100 nuclei/sample) are detailed.
- **Statistics:** Software (IBM SPSS v28, GraphPad Prism 9) and specific tests (two-way repeated measures ANOVA, Tukey’s HSD, log-rank test) are named.
  This provides clarity on the precision and validity of our approaches.

**REVIEWER COMMENT 8:**
*“Revise the order of the equations, figures, and tables throughout the whole manuscript.”*
*“The explanation of some figures is poor.”*

**OUR RESPONSE:**
We have carefully re-ordered all figures and tables to follow their first citation in the text sequentially (Figure 1, Table 1, Figure 2, Table 1A, etc.).
We have **rewritten all figure legends** to be more descriptive and informative. Explanations of what each figure shows and the key takeaways are now integrated into the main text body immediately following the figure citation.

**REVIEWER COMMENT 9:**
*“The conclusion is essential to be improved, highlight the main findings and scientific theory to support the findings.”*

**OUR RESPONSE:**
The **Conclusion section has been entirely rewritten.** It now succinctly but powerfully summarizes:

1. The core finding of temporally distinct outcomes for PARP vs. ATR inhibition.
2. The proposed mechanism (PARP inhibition protects via metabolic/oxidative stabilization, not via enhanced DSB repair).
3. The key scientific theory: **Temporal modulation is a critical determinant of DDR-targeted therapy.**
4. The translational implication for radioprotection.

**RESPONSES TO REVIEWER #2’S COMMENTS**

**REVIEWER COMMENT 1:**
*“Some grammatical errors appeared.”*

**OUR RESPONSE:**
Corrected throughout, as part of the comprehensive language edit.

**REVIEWER COMMENT 2:**
*“Please expand the results section.”*

**OUR RESPONSE:** The **Results section has been greatly expanded and is now organized into detailed subsections** (3.1.1, 3.1.2, 3.2.1, 3.2.2, 3.3.1, 3.3.2, 4.1.1, 4.1.2, 5.1, 5.2). Each subsection provides a deeper analysis of the data, includes time-course dynamics where relevant, and incorporates the direct statistical comparisons requested by the Editor. The narrative now flows logically from hematology to oxidative stress to DNA damage.

**REVIEWER COMMENT 3:**
*“Expand the introduction with some novel work.”*

**OUR RESPONSE:** As noted in response to Reviewer #1, the Introduction is now substantially expanded. We have incorporated discussion of novel concepts such as:

- The meaning of persistent γ-H2AX signaling beyond 24 hours.
- The idea of delayed DDR inhibitor intervention as a distinct strategy.
- Recent references on the metabolic roles of PARP-1 (e.g., Jagtap & Szabó, *Nat Rev Drug Discov* 2005; Andrabi et al., *PNAS* 2006).
  This sets a more novel and sophisticated stage for our study
