# Supplementary material for: Differential modulation of haematopoietic and oxidative injury by PARP-1 and ATR kinase inhibition in a murine model of acute irradiation
Source: PLoS One. 2026 Mar 6;21(3):e0344101. doi: 10.1371/journal.pone.0344101 (PMC12965550; doi:10.1371/journal.pone.0344101)
Supplement: Attachment — Submitted filename: Response to Reviewer1.docx [file pone.0344101.s004.docx]

**Response to Reviewers – PONE-D-25-44594R1**

**Manuscript Title:**
Differential Modulation of Haematopoietic and Oxidative Injury by PARP-1 and ATR Kinase Inhibition in a Murine Model of Acute Irradiation

**Corresponding Author:**
Dr. Baydaa T. Sih
Department of Physics, College of Science, University of Baghdad
Email: baydaa.sih@sc.uobaghdad.edu.iq

**Introductory Statement**

Dear Academic Editor Dr. Hesham M.H. Zakaly, and distinguished Reviewers,

We sincerely appreciate the time and expertise you have invested in evaluating our manuscript. Your insightful critiques have substantially strengthened the scientific rigor and clarity of this work. We are pleased to submit a thoroughly revised version that addresses **every comment** raised by the reviewers with precision and completeness.

All modifications are clearly visible in the marked-up manuscript using the "Track Changes" function. Below, we provide a **point-by-point response** in formal, scientifically rigorous English.

**✅ Response to Reviewer #1**

**Reviewer Comment:**

*"The authors have been adequately considering all the points made in the previous report."*

**My Response:**
We thank Reviewer #1 for their constructive engagement during the earlier round of review and are gratified that they find our previous revisions satisfactory. No further modifications were requested, and we have maintained the scientific and methodological integrity of the manuscript accordingly.

**✅ Response to Reviewer #2**

**Reviewer Comment 1 – Formatting Inconsistency (Underline):**

*"You put an underline in the conclusion section but not in other sections."*

**Our Response:**
This formatting anomaly has been rectified. The heading **"4. Conclusions"** no longer contains underlining and now adheres strictly to the uniform formatting style applied to all major section headings throughout the manuscript (i.e., **1. Introduction**, **2. Materials and Methods**, **3. Results and Discussion**, **4. Conclusions**, **5. References**). Consistency in typographic presentation has been rigorously enforced.

**Reviewer Comment 2 – Section Numbering:**

*"The conclusion and references sections are not numbered (4. Conclusion, 5. References)."*

**Our Response:**
Both sections have been correctly numbered to reflect the sequential structure of the manuscript. The document now presents:

- **4. Conclusions**
- **5. References**

This adjustment ensures logical continuity and compliance with standard manuscript formatting conventions.

**Reviewer Comment 3 – Disorganization of Materials and Methods:**

*"Why is the materials and methods section not organized? there is not suitable table, etc."*

**Our Response:**
The **Materials and Methods** section has been **completely restructured** to achieve optimal clarity and reproducibility. It is now systematically divided into **eleven logically ordered subsections**, enumerated from **2.1 to 2.11**:

- 2.1. Study Design and Temporal Rationale
- 2.2. Animals, Ethics, and Husbandry
- 2.3. Irradiation Procedure
- 2.4. Post-Irradiation Pharmacological Interventions
- 2.5. Experimental Groups
- 2.6. Longitudinal Monitoring and Survival Analysis
- 2.7. Multi-Timepoint Biospecimen Collection
- 2.8. Haematological Analysis
- 2.9. Oxidative Stress and Antioxidant Capacity
- 2.10. DNA Damage Assessment and Target Engagement Validation
- 2.11. Statistical Analysis

Furthermore, a **comprehensive summary table**—**Supplementary Table S1**—has been constructed and integrated. This table provides a complete, at-a-glance overview of all eight experimental groups, including irradiation status, pharmacological treatments, doses, routes of administration, timing, animal numbers per time point, and total cohort sizes. This addition directly addresses the reviewer's concern and substantially enhances the methodological transparency of the study.

**Reviewer Comment 4 – Omission of Animal Images:**

*[Implied by request for organization and table; explicitly addressed by addition of Figure 1.]*

**Our Response:**
A dedicated subsection (**2.2. Animals, Ethics, and Husbandry**) now consolidates **all animal-related information**, including ethical approval references, housing conditions, acclimatization, randomization, humane endpoints, and euthanasia protocols. Crucially, we have explicitly cited:

*"Figure 1 illustrates representative images of the animals in the housing facility and during experimental procedures."*

This figure has been embedded within the manuscript to provide visual documentation of animal welfare and experimental conditions, thereby fulfilling the highest standards of ethical reporting.

**Reviewer Comment 5 – Reference List Deficiencies:**

*[Implied by Journal Requirement 2: "review your reference list to ensure that it is complete and correct."]*

**Our Response:**
The reference list has undergone a **comprehensive and rigorous revision**:

- All entries have been meticulously formatted according to **PLOS ONE's Vancouver-style conventions**:
  ✓ Abbreviated journal titles (indexed in PubMed/Medline)
  ✓ Complete and correctly punctuated DOIs
  ✓ No italicization of titles or journal names
  ✓ Correct volume, issue (where applicable), and page ranges
- **Temporal currency**: Of the 50 references, **36 (72%)** are from the period **2023–2026**, ensuring that the manuscript engages with the most contemporary evidence. Seminal foundational works (e.g., Jackson & Bartek 2009, Rogakou 1998) have been retained where historically indispensable.
- **Verification of retractions**: Each cited publication has been cross-verified against the Retraction Watch database and PubMed. **No retracted articles** are included in the reference list.
- **Supplementary citations**: No external recommendations for citation were mandated by the editor or reviewers; therefore, the reference list remains exclusively composed of works directly pertinent to the study.

**Reviewer Comment 6 – Language and Proofreading:**

*"The volume of minor errors indicates that the manuscript required more thorough proofreading before resubmission."*

**Our Response:**
The entire manuscript has undergone an **exhaustive linguistic and stylistic revision** supervised by a senior researcher with extensive experience in biomedical publication. The following actions were executed:

- Conversion of all procedural descriptions to **passive voice** in accordance with scientific writing conventions (e.g., "was performed," "were administered," "was quantified").
- Elimination of anthropomorphic and colloquial expressions.
- Correction of typographical, grammatical, and punctuation errors throughout the text.
- Removal of all extraneous non-printing characters, including residual HTML/RTL markup (e.g., [dir="rtl"], {dir="rtl}), which were artifacts of cross-platform document conversion.

The manuscript now exhibits **professional-grade scientific English** and is free of distracting mechanical errors.

**Summary of Major Revisions Executed**

| Aspect | Original Status | Revised Status |
| --- | --- | --- |
| Conclusion heading formatting | Underlined | Plain text, uniform with other headings |
| Section numbering | Incomplete (Conclusion, References unnumbered) | Consecutive: 4. Conclusions, 5. References |
| Materials and Methods organization | Unstructured prose | 11 clearly delineated subsections (2.1–2.11) |
| Experimental groups summary | Absent | **Supplementary Table S1** (full details) |
| Animal ethics & Figure 1 | No explicit figure citation | Explicit citation; images embedded |
| Reference list currency | Majority pre-2020 | 36/50 references (2023–2026) |
| Reference formatting | Italicized, incomplete DOIs | PLOS ONE style, full DOIs, no italics |
| Language quality | Passive/active inconsistency, typographical errors | Fully proofread, passive voice, error-free |
| Hidden markup | [dir="rtl"], {dir="rtl} tags present | Completely removed |

**Concluding Statement**

We trust that this revised manuscript now meets the **rigorous publication standards** of *PLOS ONE*. All concerns raised by the reviewers have been addressed with **thoroughness and scholarly integrity**. No additional experiments or data were requested, and we confirm that the scientific conclusions remain robust and fully supported by the evidence presented.

We are grateful for the opportunity to improve our work through this process and respectfully submit this revision for your final consideration.

**Sincerely,**

**Baydaa T. Sih, Ph.D.**
Corresponding Author
Department of Physics, College of Science
University of Baghdad, Al-Jaderyia Campus, Baghdad, Iraq
Email: baydaa.sih@sc.uobaghdad.edu.iq

**Date:** February 11, 2026
